# Application of Survival Analysis and Multistate Modeling to Understand Animal Behavior: Examples from Guide Dogs

**DOI:** 10.3389/fvets.2017.00116

**Published:** 2017-07-28

**Authors:** Lucy Asher, Naomi D. Harvey, Martin Green, Gary C. W. England

**Affiliations:** ^1^School of Veterinary Medicine and Science, University of Nottingham, Leicestershire, United Kingdom; ^2^Centre for Behaviour and Evolution, Newcastle University, Newcastle, United Kingdom

**Keywords:** epidemiology, survival analysis, multistate models, guide dogs, animal behavior

## Abstract

Epidemiology is the study of patterns of health-related states or events in populations. Statistical models developed for epidemiology could be usefully applied to behavioral states or events. The aim of this study is to present the application of epidemiological statistics to understand animal behavior where discrete outcomes are of interest, using data from guide dogs to illustrate. Specifically, survival analysis and multistate modeling are applied to data on guide dogs comparing dogs that completed training and qualified as a guide dog, to those that were withdrawn from the training program. Survival analysis allows the time to (or between) a binary event(s) and the probability of the event occurring at or beyond a specified time point. Survival analysis, using a Cox proportional hazards model, was used to examine the time taken to withdraw a dog from training. Sex, breed, and other factors affected time to withdrawal. Bitches were withdrawn faster than dogs, Labradors were withdrawn faster, and Labrador × Golden Retrievers slower, than Golden Retriever × Labradors; and dogs not bred by Guide Dogs were withdrawn faster than those bred by Guide Dogs. Multistate modeling (MSM) can be used as an extension of survival analysis to incorporate more than two discrete events or states. Multistate models were used to investigate transitions between states of training to qualification as a guide dog or behavioral withdrawal, and from qualification as a guide dog to behavioral withdrawal. Sex, breed (with purebred Labradors and Golden retrievers differing from F1 crosses), and bred by Guide Dogs or not, effected movements between states. We postulate that survival analysis and MSM could be applied to a wide range of behavioral data and key examples are provided.

## Introduction

Epidemiology is the study of patterns of health-related states or events in populations. The discipline has been dominated by statistics, which has resulted in a wide and flexible range of statistical methods developed in response to the particular challenges and types of data. For example, statistical epidemiological methods have been designed to cope with discrete data, control for (but not ignore) differences between individuals and groupings of individuals and to consider time to, or between, discrete outcomes (and dependence on previous state). The statistical methods developed can usefully be applied to the analysis of behavioral data. Here, we demonstrate this by applying two popular epidemiological models, survival analysis and multistate modeling (MSM), to data on guide dog behavior.

In the study of health, many outcomes of interest are discrete. For example, does a patient have a disease or not? do they die from a disease or not? is the patient susceptible, infected, or recovered from an infectious disease? Similarly, categorical data are common in the study of behavior. For example, does the subject learn a trained task? Does a subject fight its competitor or not? Does the subject choose option a, b, or c in a choice test? A further commonality between behavior and health outcomes is that time is often an important aspect of the outcome. An epidemiologist might study whether or not a patient died and how long this took, whereas an ethologist may study whether and animal learned a task and how many trials this took. A final similarity is a lack of heterogeneity between subjects of interest. People from the same family or household may have a shared susceptibility to a disease event and animals’ from the same litter, or held in the same pen, may have a shared susceptibility to a behavioral event. Such similarities between types of data have resulted in some exploitation of similar statistical methods in both disciplines: random effects models, which account for unobserved heterogeneity, are common in both disciplines (1–4). However, statistical methods designed to study health-based events have been underutilized in behavior.

One common epidemiological statistical method is survival analysis. Survival analysis, as the name suggests, was developed to examine mortality data and risks associated with time until death. Death is a discrete (binary) outcome. Using survival analysis, it is possible to model: a time variable, which is the time spent in a given state or the time between two events; a survival function, which is the probability of an individual surviving beyond time *t*; and a hazard function, which represents the probability that an individual “alive” at time *t* experiences “death” in the next period, *t* + 1. Frailty models can be used to account for a lack of heterogeneity between subjects. Survival models also allow for censoring where the outcome is not recorded at a point in time for a particular subject. Survival models may be particularly useful for studying behavior and have to date been applied to a limited range of behavioral data including: cognitive and judgment bias tasks (5); choice/preference tests (6); latency to perform behavioral response (7, 8); and time taken for pets to be rehomed (9).

Survival analysis, typically has a binary outcome, but MSM can be extended to incorporate more than two discrete events or states. The archetypical use of MSM is to understand transitions, and the timing of transitions, between disease states. For example, when studying cancer, epidemiologists have considered the rate at which patients move between states of treatment for cancer, remission, and death (10). The multistate model accounts for dependencies of timings of subsequent events and dependencies of timings of competing events. Like survival analysis, censoring and frailty models can be used. MSMs provide a range of possible options for modeling the dependence of the transition rates on time. Models can be: time homogeneous models, which consider transitions to be constant and independent of time, Markov models where transitions depend on current state; and semi-Markov models where transitions depend on the current state but also on the entry time into the state. To date, MSM have rarely been applied to the behavioral data, with one exception being their application to understand transitions between mobility scores in livestock (11).

To illustrate the application of epidemiological techniques to the analysis of animal behavior data, we use three previously published datasets from Guide Dogs. Guide Dogs UK are the largest breeder and trainer of dogs in the UK and have been keeping detailed records on behavior and health of dogs they breed and train for many decades. Retrospective analysis of these data has proved useful in studying patterns of health (12) and behavior (13). In addition, in recent years, Guide Dogs has been prospectively collecting more detailed behavioral data on dogs during training to better understand how to monitor and record behavior (14–16). The majority of potential guide dogs are bred internally, but a minority is purchased from breeders. All are placed with a volunteer puppy walker for the phase of Puppy Walking (obedience, socialization, and habituation), when they are approximately 6–8 weeks of age. When dogs reach approximately 12–14 months of age, and they are deemed ready, they will begin Guide Dog training during which time they are trained to guide a blind or partially sighted individual, typically in two stages by two trainers. In the last few weeks of this training, dogs are matched with a guide dog owner and the dog and owner are trained together. Dogs, which are successful in completing training are described as qualified, but those deemed unsuitable can be withdrawn from training at any stage. Here, as an example of how epidemiological methods can be used to provide insights into animal behavior research, we apply survival analysis and MSM to Guide Dogs data, considering the factors, which are associated with a dog qualifying or not, movements between different stages of training and the timing of both of these.

The three datasets considered in this study have all been previously analyzed using alternative (more standard) statistical methods and the results published elsewhere (13, 15, 17). In brief, previously, we presented controlled observations of 6- to 8-week-old puppy behavior, the Puppy Profiling Assessment (PPA), where three (stroking, fake prey, ramp) of seven stimuli presented (also including following, retrieve, gentle restraint, noise, tunnel) could be usefully combined and associated with whether dogs completed guide dogs training to become a working guide dog (success) (17). Reactions to these stimuli could be applied to identify a small number of dogs that were later withdrawn from guide dog training with high specificity but low sensitivity, and a subsequent study suggested responses to stimuli were heritable (18). Using a questionnaire approach, completed by Guide Dog staff for trainee guide dogs of 5, 8, and 12 months of age, seven behavior traits were identified, adaptability; body sensitivity; distractibility; excitability; general anxiety; trainability and stair anxiety, which all showed associations with later success in guide dog training (15). Together, the traits could be applied to early identify a small proportion of dogs with high specificity that were later withdrawn and which were successful. A final relevant publication considered dogs withdrawn from service as a guide dog for behavior reasons based on retrospective data (13). We found that there was no peak in working life in which dogs were withdrawn for behavior, and sex and breed were important factors in age of behavioral withdrawal. In all cases, the statistical methods used to explore the association with later success in guide dog training or withdrawal from training or working were based on variants of logistic regression models. Here, we extend analysis from these three studies to include the entire span of a guide dog from training to retirement for the retrospective dataset [hence overlapping but not identical data between this paper and (13)]. The aim is to illustrate the utility of survival analysis and multistate modeling and to explore the additional insights these methods provide beyond more traditional methods of analysis.

## Methods

### Behavior Data

We used three datasets for this analysis (see Table 1): (1) retrospective data spanning 10 years (dogs born 2000–2011), (2) data collected on behavior of 6- to 8-week-old puppies in a controlled behavior test named the PPA (dogs born between March 2006 and February 2007), and (3) data collected using a validated questionnaire on dog behavior during puppy walking (dogs born between 6/12/2011 and 1/1/2013).

**Table 1:** Descriptions of three datasets used for analysis and list of outcome variables and predictors considered.

Datasets for analysis	*N* (M:F)	Outcome variable(s)	Predictors
Retrospective Guide Dogs data	10,968 (5,703M: 5,265F)	Survival analysis: time taken to withdrawal from training or working for behavioral reasons	Breed (3,417 Golden Retriever × Labrador, 3,387 Labrador, 790 Labrador × Golden Retriever, 3,429 “Other” 11 breeds or crosses), Sex, Guide Dogs bred (9,873 bred by Guide Dogs and 1,095 bred externally)
		MSM: transitions between “Puppy Walking,” “Training,” and “Withdrawal” from training	

Controlled behavior test: puppy Puppy Profiling Assessment (PPA) data	801 (421M: 380F)	Survival analysis: time taken to withdrawal for behavior reasons from training	Behavior test scores on seven point scale for: following-response, retrieve-response, restraint-response, restraint-recovery, noise-response, stroking-response, stroking-recovery, squirrel-response, squirrel-recovery, tunnel-response, ramp-response
		MSM: transitions between “Puppy Walking,” “Training,” and “Withdrawal” from training	

Behavior questionnaire of dogs during puppy walking	1,402 (678M: 724F)	Survival analysis: time taken to withdrawal for behavior reasons from training	Trait scores from questionnaire: distractibility, excitability, trainability, general anxiety, adaptability, body sensitivity and stair anxiety Mean score of neutral body posture
		MSM: transitions between “Puppy Walking,” “Training,” and “Withdrawal” from training	

Retrospective data were used to gain basic information on withdrawal from guide dog training or working life. Data were accessed in October 2014 from Guide Dogs record system. The training outcome of dogs at that time was recorded.

In the controlled behavior test, puppies were presented with a series of controlled stimuli and their reactions were scored by a member of Guide Dogs staff on a seven point scale according to behavioral descriptors. These tests took place from May to October 2012. The later outcome of dogs (withdrawal or qualification and movements between stages) was accessed from Guide Dogs records in October 2014, but these decisions were made independently of scores on the PPA. Scores of the PPA have previously been found to be associated with qualification as a guide dog, supporting the predictive criterion validity of the test (17). Some scores on the PPA are also heritable (18) and Guide Dogs use this test routinely to record behavior of puppies. In the PPA, puppies are scored according to their response or recovery to stimuli and the human assessor. Full details can be found in Asher et al. (17), but in brief, the stimuli the puppies encounter are: “Following” where they are encouraged to follow a person, a toy, which they are encouraged to “Retrieve,” a brief and gentle “Restraint,” playback of aircraft “Noise,” “Stroking” by a person, a small furry object moved fast on a string to mimic a “Squirrel”-like prey, a “Tunnel,” which they are encouraged to move through, and a “Ramp,” which they are encouraged to walk over.

Five of these stimuli are scored according to a dog’s immediate reaction only (following, restraint, noise, tunnel, ramp), and three are scored according to a dog’s immediate reaction and subsequent recovery reaction (retrieve, stroking, squirrel). This results in a total of 11 scores, each scored from 1 to 7. The dogs tested came from 11 breeds or crossbreeds of Golden Retrievers, Labradors, German Shepherd dogs, and Flat coat retrievers. The most common breeds were: Labradors (260) and Golden retriever × Labradors (205). The majority of dogs were bred by Guide Dogs (791/801).

**Figure 1:**
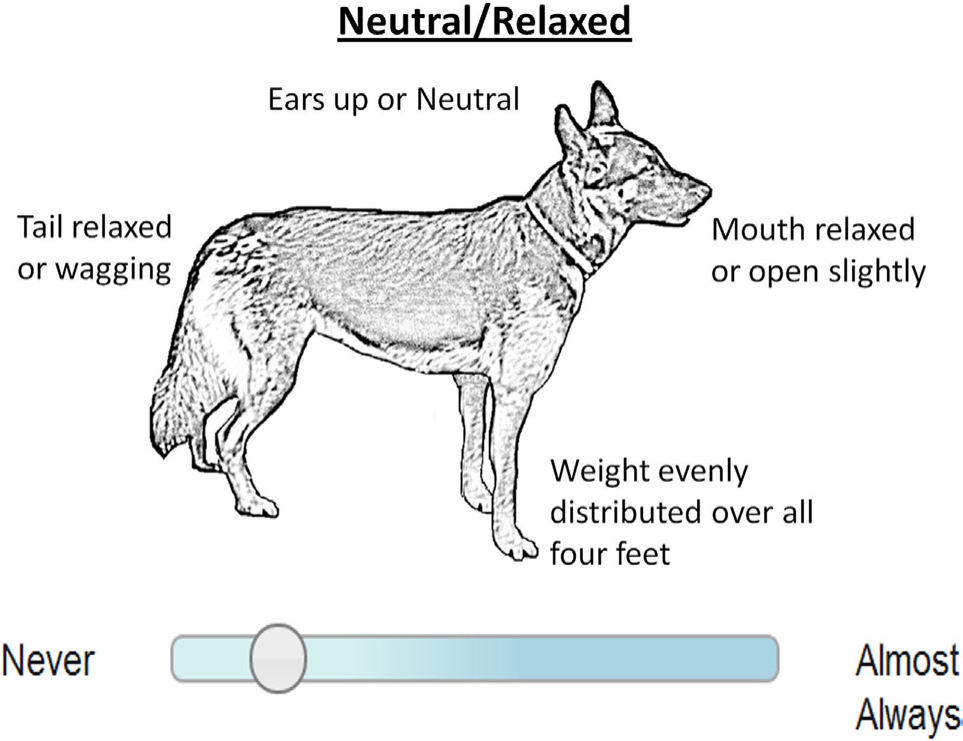
Neutral image and visual analog scale used to score behavior in questionnaire about 5-month-old potential guide dogs.

In the questionnaires, Guide Dogs staff responsible for supervising a dog’s progress during the Puppy Walking stage of training (Puppy Training Supervisors) were asked to complete a series of questions about the dog’s behavior scored on a visual analog scale (15). Data used here were collected when the dogs were 5 months of age (plus or minus 1 week) for all dogs in Guide Dogs Puppy Walking scheme from 6/12/2011 to 1/1/2013 (*n* = 1,401). Information on the later outcome (withdrawal or qualification and movements between stages) was retrieved from Guide Dogs records in October 2014. Outcome and movement decisions were made independently of questionnaire scores, which were not provided to Guide Dogs staff. The questions were grouped into traits and the means of these questions formed a score for each trait. This provided trait scores with the following names: distractibility, excitability, trainability, general anxiety, adaptability, body sensitivity and stair anxiety. Trialed alongside the questionnaire were postural images of dogs appearing “Neutral,” scored in five different situations. Puppy Training Supervisors were asked to rate how often the dog appeared to show body language illustrated in the neutral image using a visual analog scale from Never to Almost Always (see Figure 1) for each of the five different situations (when…it encounters another dog, …stranger(s) advance directly toward to dog, …a child/children advance directly toward the dog, …unfamiliar visitors approach the car or its kennel, …approached in a confined space) and a mean was calculated across these.

The questionnaire was developed using a combination of etic and emic approaches. It was tested to confirm internal reliability construct validity, concurrent criterion validity (using behavior observed in a separate controlled behavior test), rank-order consistency over time (to confirm that trait scores represented temporally stable traits), and was associated with qualification as a guide dog, supporting predictive criterion validity (15). Dogs were from ten breeds and crossbreeds of Golden Retrievers, Labradors, German Shepherd dogs, and Flat coat retrievers. The most common breeds were: Golden Retriever × Labrador (474), Labrador (407), Golden Retriever (131), Labrador × Golden Retriever (132). The majority were bred by Guide Dogs (1,362/1,402).

### Statistical Methods

Survival analysis was completed using a Cox proportional hazard model implemented in the R package “survival.” A frailty term for Dam included in all models and a frailty term of assessor (ID of the person who completed the questionnaire) was included in the behavior questionnaire models. For these retrospective data, we used the age at which dogs were withdrawn permanently from training or working for behavior reasons as the outcome variable. Dogs that qualified could reach retirement and be retired at approximately 8–10 years of age or be withdrawn prior to this for behavior, health, or guide dog owner reasons. For the purposes of this, dataset dogs withdrawn for health or guide dog owner reasons were excluded from analysis. The survival curve of dogs withdrawn for behavioral reasons was plotted against dogs that retired or were censored (included in time series until the point when data were not available). The effects of breed, sex, and whether or not the dog was bred by Guide Dogs (see Table 1) were then considered on this outcome variable in univariate Cox proportional hazards models. Finally dogs’ time of movement from training to qualification was considered in comparison to dogs withdrawn for behavioral reasons using survival curves, for illustrative purposes. For data from the controlled behavior test and the behavioral questionnaire, we considered the association between the behavioral measures taken using each of these methods and subsequent withdrawal from training for behavior reasons (see Table 1). A covariate of age was applied if needed based on the assumption of proportional hazards, which was tested using the function cox.zph. For each survival analysis completed, significant results are presented with hazard ratios (HRs), 95% confidence intervals (CIs), *p*-values (*p*), and median survival times for the time taken to withdrawal.

Multistate models were used to analyze transitions between states of training and working assuming next the state was dependent on time in current state (Semi-Markov model). The models were implemented in the “Epi” and “splines” packages in R and included frailty terms for the Dam. For the retrospective data effects of sex, breed, and Guide Dogs bred on transitions between dogs in training, qualified, and withdrawn for behavior reasons were modeled. Qualified dogs were those working with a guide dog owner. Dogs, which were withdrawn for health or guide dog owner reasons were censored prior to the withdrawals. For the controlled behavior, PPA test and the behavior questionnaire data, transitions between Puppy Walking, Training, and Withdrawal were considered. The effects of the 11 PPA scores, and seven trait scores and postural image scores of the neutral posture score from the questionnaire were considered in two separate MSMs. The number of transitions between states and total dog years spent in each state are presented. Significant effects of predictors are presented with odds ratios, CIs, and *p*-values presented for effects of the probability of movement between states and medians, IQR, and *p*-values presented for the time taken to move between states.

The criteria for accepting significance were *p* < 0.05.

## Results

### Survival Analysis

**Figure 2:**
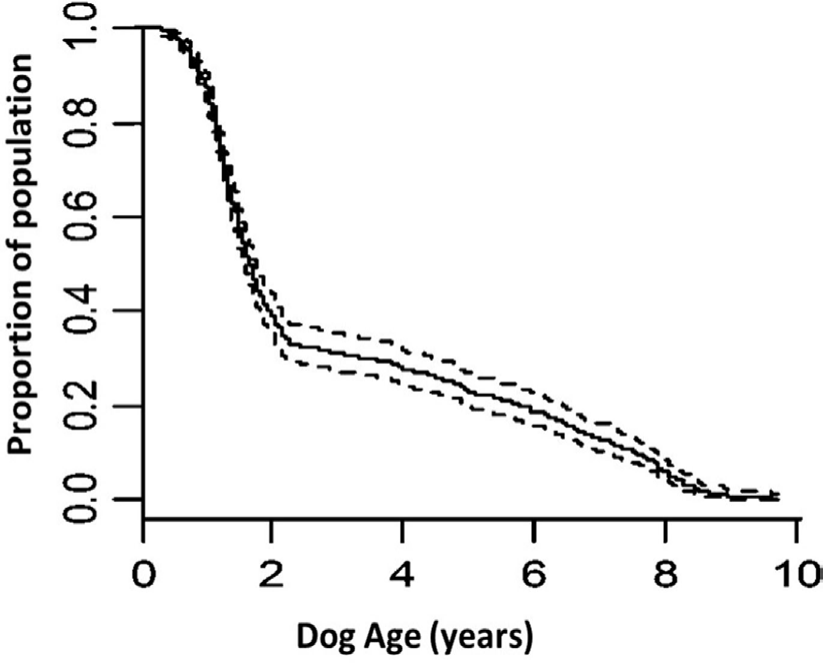
Survival curve of the entire guide dogs span for withdrawn from training and working for behavioral reasons (dotted line represents confidence intervals).

**Figure 3:**
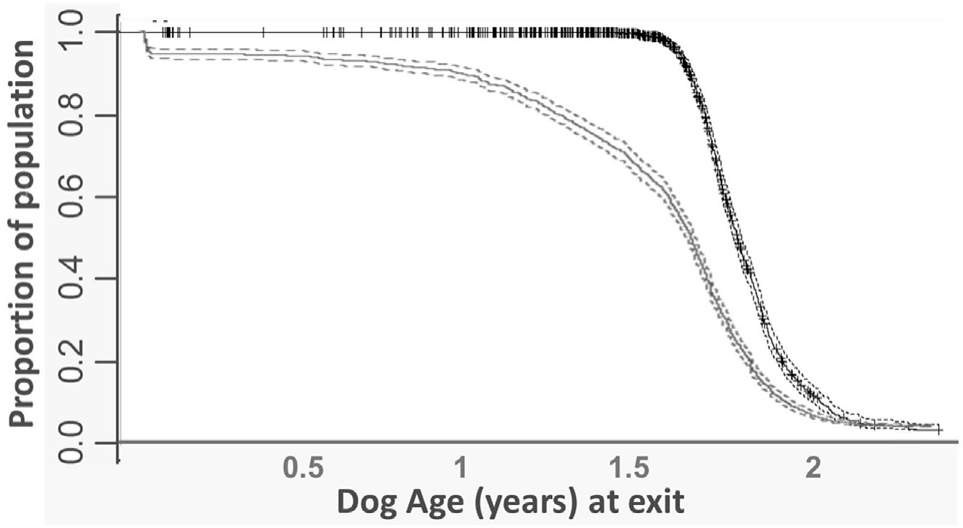
Survival curves focused on training phase of guide dogs span, for all dogs exiting training for any reason (black line) and dogs exiting training due to behavioral withdrawals (dotted line represents confidence intervals).

Using retrospective data, there were 4,429 withdrawals for behavior during training or working. Of these withdrawals, 2,085 were females, 2,344 were males; 3,695 were bred by Guide Dogs and 734 were bred externally; and 1,055 were Golden retriever × Labradors, 1,591 were Labradors and 235 were (sire × dam) Labrador × Golden retrievers. For dogs withdrawn for behavioral reasons from training or working life, the most common time period for this withdrawal was during training (the first 2 years of life, see Figure 2). Following an initially steep survival curve during the first 2 years of life, a steady slope was found with dogs withdrawn for behavior at approximately an equal rate from 2+ until the age of 8.5 years of age, when many dogs were retired from working life. Focusing on the training period, most dogs withdrawn for behavioral reasons were withdrawn between 1 and 1.5 years of age (Figure 3), whereas dogs, which moved out of training for other reasons, such as qualifying as a guide dog did so at 1.5–2 years of age. The time prior to withdrawal was influenced by sex, breed, and whether dogs were internally bred. Labradors were withdrawn faster (by a median of 2 weeks, HR: 1.09 CI: 1.01–1.18, *p* = 0.026) and Labrador × Golden Retrievers slower (by a median of 2.5 weeks) than Golden Retriever × Labradors (by a median of 1.5 weeks, HR: 0.83, CI: 0.72–0.97, *p* = 0.014). The *R* squared of this model was 0.05 (where *R* squared indicates the proportion of the variance in the dependent variable that is attributable to the variables in the model). Dogs not bred by Guide Dogs were withdrawn faster than those bred by Guide Dogs (3.1 weeks, HR: 1.13 CI: 1.04–1.23, *p* = 0.002). The *R* squared was 0.02. Males were withdrawn faster than females by 4.2 weeks (HR: 1.26 CI: 1.19–1.34, *p* < 0.001). The *R* squared was 0.013.

From the controlled behavior test set, 289 dogs were withdrawn from training. One of the 11 scores of behavior from this test of puppies at 6–8 weeks of age was associated with probability of withdrawal from training for behavioral reasons. Puppies that were scored higher on as recovering after exposure to the Squirrel stimuli were less likely to be withdrawn (HR: 0.81, CI: 0.69–0.94, *p* = 0.0047). However, dogs that scored higher on this element of the test were withdrawn a median of 1.24 weeks later. The frailty term of Dam was important in this model (chi-squared = 543, df = 76.9, *p* < 0.001). In this case, the inclusion or not of the frailty term had little influence, either on the estimate or the significance of the fixed effects.

From the behavior questionnaire dataset, 548 dogs were withdrawn from training. Dogs with higher scores of excitability were withdrawn faster by a median of 2.15 weeks and were more likely to be withdrawn (HR = 1.71, CI: 1.05–2.78, *p* = 0.03). Dogs scored as neutral in body posture more often were less likely to withdrawn (HR = 0.58, CI: 0.38–0.88, *p* = 0.009). Assessor was not needed as a frailty term and was, therefore, removed from the model. The frailty term of Dam was important in the model (chi-squared = 2,555, df = 174.9, *p* < 0.001). In this case, the inclusion of the frailty term changed the model such that excitability and neutral would not have been significant without the frailty term.

### Multistate Modeling

**Figure 4:**
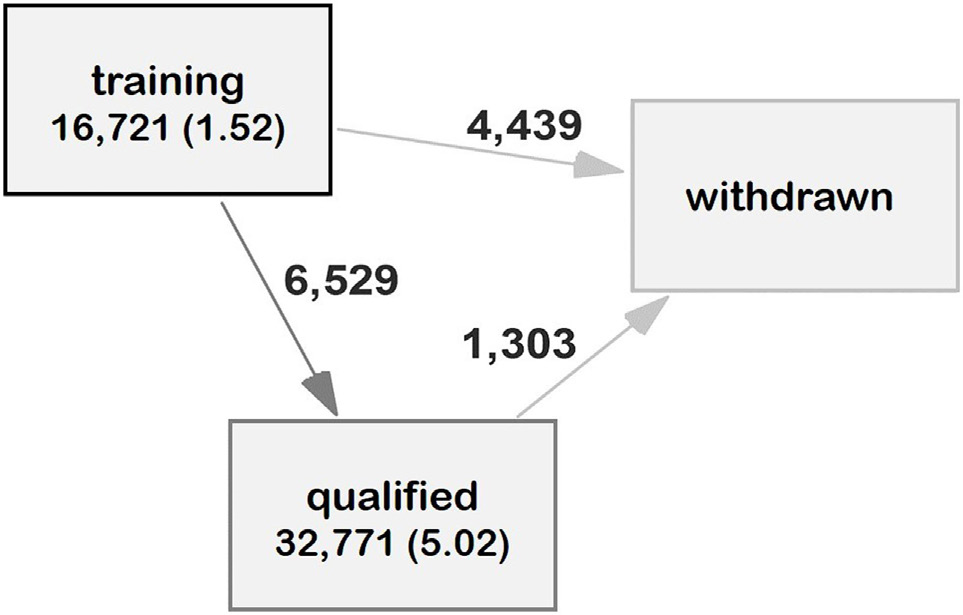
Multistate model of transitions between states of guide dog training and working from retrospective data. The arrows indicate the direction of movements between states and the numbers on the arrows the number of dogs moving between states. The total dog years spent in each state across the dataset is written in each state box, with the mean years per dog in brackets.

Using retrospective data, it appeared that the majority of dogs (6,529/10,968) transitioned from training to qualification and most of these were never withdrawn from working for behavioral reasons (see Figure 4). Dogs were a median of 1.68 years when they moved from training to being qualified. Transitions between training and withdrawal for behavior reasons were associated with sex, breed, and bred by Guide Dogs. Dogs were withdrawn faster than bitches (by 5 weeks, HR: 1.16 CI: 1.10–1.23, *p* < 0.001). Golden retriever × Labradors were withdrawn at a median age of 1.75, which did not differ from Labrador × Golden retrievers (median 1.62). All other breeds took less time before moving to withdrawn for behavior (Labradors, median 1.44, HR: 1.45 CI: 1.34–1.56, *z* = 9.25, *p* < 0.001; Golden Retrievers median = 1.42, HR: 1.53 CI: 1.38–1.70, *p* < 0.001, and other breeds median 1.41 HR: 1.58, CI: 1.44–1.71, *p* < 0.001). Dogs bred by Guide Dogs spent longer in training before movements to withdrawal for behavior (by a median of 10 weeks, HR: 1.68, CI: 1.56–1.82, *p* < 0.001). Breed and sex were associated with transitions from training to qualified: bitches took longer to move from training to qualified (median for bitches 1.78 years and dogs 1.69 years, HR: 1.49, CI: 1.30–1.70, *p* < 0.001). The Other breed group (median age 1.79, HR: 0.63, CI: 0.52–0.76, *p* < 0.001) and Labrador × Golden retrievers (median age 1.79, HR: 0.63, CI: 0.50–0.85, *p* < 0.002), took longer to qualify than Golden retriever × Labrador (median age: 1.70). Transitions between qualified and withdrawn for behavioral reasons were associated with breed but not sex and whether dogs were bred by Guide Dogs or not. The Other breed groups were qualified for less time (median of 3.87 years) than the reference breed of Golden retriever × Labrador (median of 4.26 years, HR: 1.43, CI: 1.18–1.74).

From the controlled behavior test, most dogs transitioned from puppy walking to training (646), with 66 withdrawn before entry to training and 191 withdrawn after entry to training. Dogs, which retrieved a toy more spent less time training (HR: 1.33, CI: 1.13–1.56, *p* < 0.001 by a median of 5 weeks) before being withdrawn and dogs, which responded less to a person after encountering a Squirrel-like moving object were withdrawn from puppy walking at a faster rate (HR: 0.77, CI: 0.61–0.97, *p* = 0.025, by a median of 2 weeks from highest to lowest scores). Dogs that reacted more to the Squirrel like object (HR: 0.90, CI: 0.82–0.99, *p* = 0.047, by a median of 1 week from lowest to highest scores) and dogs, which retrieved a toy less (HR: 0.91, CI: 0.83–0.99, *p* = 0.036, by a median of 2 weeks from highest to lowest scores) were slower to move into training.

**Figure 5:**
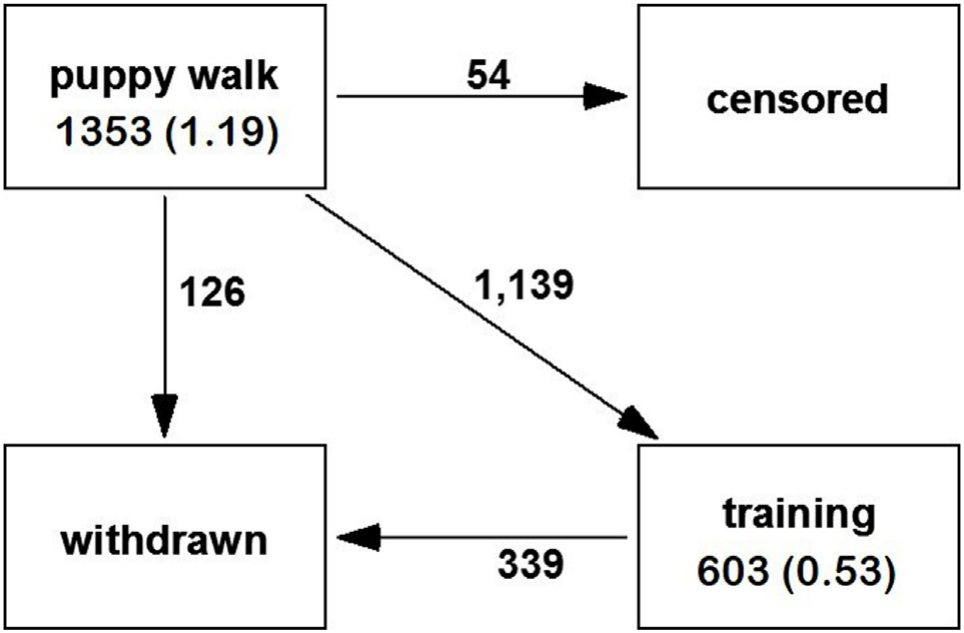
Multistate model of transitions between states of guide dog training, from behavioral questionnaire data. The arrows indicate the direction of movements between states and the numbers on the arrows the number of dogs moving between states. The total dog years spent in each state across the dataset is written in each state box, with the mean years per dog in brackets.

**Figure 6:**
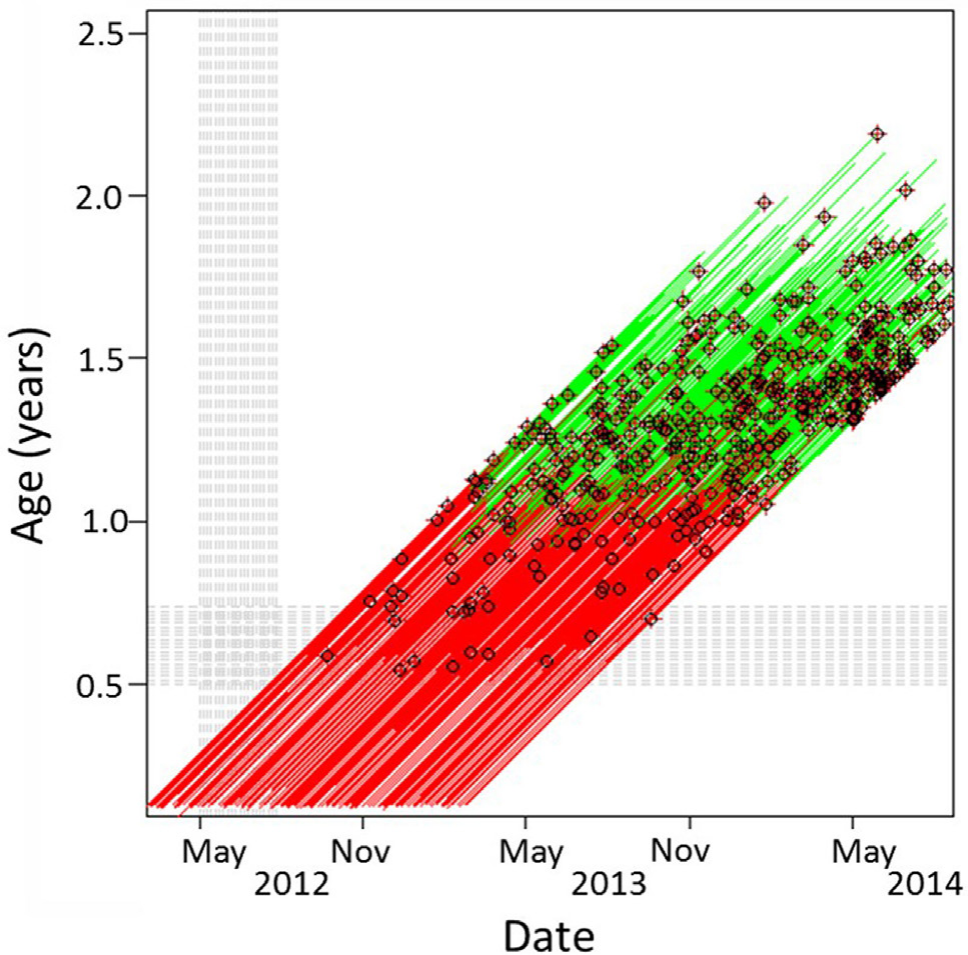
Age and date of dogs in guide dog training, with stage and exits indicated from the behavioral questionnaire sample. Each line represents one dog. Red lines show dogs in puppy walking, green lines show dogs in training, black dots show dogs exiting training for behavioral reasons, with the absence of black dots indicating continuation within Guide Dogs.

From the behavior questionnaire, the most common transition was between puppy walking and training, with fewer dogs moving to the withdrawn state (Figure 5). Dogs spent twice as long in puppy walking than they did in training. Most withdrawals for behavior reasons occurred during training (Figure 6). Dogs scored higher on the trait Excitability transitioned to training from puppy walking faster (HR = 3.29, 1.62–4.45, *p* < 0.001, by a median of 0.7 weeks from high to low scores). General anxiety was associated with movements between puppy walking and withdrawal for behavioral reasons, with more anxious dogs moving at a faster rate between these states (HR: 5.78, CI: 1.28–25.62, *p* = 0.022, by up to 7 weeks). Dogs that were more anxious (on the General Anxiety scale) stayed longer in puppy walking (HR: 0.53, CI: 0.32–0.85, *p* = 0.009, by 1.4 weeks) than dogs that scored lower on these traits.

## Discussion

The value of using survival analysis and MSM techniques, more often applied to analyze patterns in health data, to understand animal behavior has been demonstrated utilizing data from guide dogs. Survival curves showed that if guide dogs were going to be withdrawn for behaviour reasons this occured primarily before they had qualified. After qualification, there was a steady rate in dogs withdrawn for behavior reasons, which fits with previous findings from our group using alternative methods (13). The exact numbers of dogs transitioning between stages is not shown by our data due to exclusion of dogs, which were withdrawn for guide dog owner or health reasons before reaching retirement (~20% of all dogs). Rather, these examples of use of epidemiological methods illustrate patterns of timings and effects of key explanatory variables. Survival analysis allowed modeling of the entire training and working life of a guide dog, which previously has been modeled as two outcome variables: whether a dog completed guide dog training to work with a client (or not) (15); and the lifespan of a working guide dog (13). Modeling these two outcomes together provides a better overview and reduces the number of analyses that need to be undertaken, thereby potentially reducing type 1 errors. The MSM perhaps provided the most novel information compared to previous analysis on these data. The multistate models revealed how many years of dog training (including the time spent in the home of a puppy walker and more formal guide dog training) was required for each year of dog working (approximately 1:2). The timings of movements between stages of guide dog training were different for dogs scored as more anxious or excitable. Finding that some types of dogs move more quickly through training than others could be used to help identify the characteristics of dogs that suited to shorter training times. From an applied perspective, length of training has an associated cost and there have been differences found at breed level for the effective length of training in dogs (19). The added value of using the MSM compared to previous findings shown here serves to highlight the potential it could have for other areas of studying animal behavior. Since behavior is frequently recorded in terms of behavioral states, there could be benefit in the use of MSM to model the timing and probability of movement between states.

Basic dog factors such as the sex, breed, and whether dogs were Guide Dog bred affected either the probability of withdrawal for a behavior reason [in keeping with previous findings (19–21)] or how long it was before dogs were withdrawn. The new findings on the length of time until withdrawal are important since they could inform training practices within Guide Dogs. When these data were collected, it was typical for most dogs that were withdrawn from training to be withdrawn between 1 and 1.5 years of age. Data here from a controlled behavior test and a questionnaire of dog behavior suggest that at least some dogs, which were later withdrawn for behavior could be identified at 6–8 weeks or 5 months of age, respectively. It is expensive to train a guide dog and these costs increase with progression in training. Thus, earlier identification of dogs, which are later withdrawn could result in large savings to Guide Dogs.

One of the eleven scores assigned to dogs during a controlled puppy test, the PPA, was associated with survival and one with the time in training in MSM. Previously and in a separate dataset, these scores of response to a Squirrel-like moving object and retrieval of a toy were associated with later success in training using a logistic regression approach (17). Retrieval of objects in young dogs seems indicative of their ability to work cooperatively with people (22, 23). Scores on the retrieval element of the PPA have previously been found to be heritable above chance levels (18), suggesting this behavior could be selected for. In this study, Dam was an important effect in the survival models, which is further suggestion that behavior measured in this test could be heritable, or at least responsive to Dam environment.

From behavioral questionnaires of 5-month-old dogs, those with high scores on a trait named “Excitability” were more likely to be withdrawn for behavioral reasons. More excitable dogs were also moved more quickly between puppy walking (socialization and basic obedience stage of training) and the more structured guide dog training. The former finding supports previous research from our group (15), but the latter is novel. It is possible that dogs that are excitable may be moved to training faster than less excitable dogs in an attempt to provide additional support and structure. Two other scores on the behavioral questionnaire were associated with survival in training rather than withdrawal for behavioral reasons or transitions between states of puppy walking, training, and withdrawal: a trait named General Anxiety and higher scores of Neutral body posture. While anxiety behavior has previously been associated with withdrawals from guide dog training (15, 24–26), questions about neutral body posture have not previously been used to understand dog behavior. Scoring neutral posture using the method applied in this study also combines information on the initial arousal response, the time taken to recover from the arousal, and generalization of the response across contexts. Dogs showing neutral posture more often are also showing an absence of signs of high arousal of either positive (e.g., excitement, distraction) or negative (stress, anxiety, fear, aggression) valence, more often. There is evidence that people find states such as aggression (27) difficult to recognize in dogs, so, a focus on neutral posture could offer a useful alternative approach.

Survival analysis is a flexible statistical tool, which has been applied to some types of behavior data, perhaps, most notably, data on the length and outcome of animal contests (7). However, the approach could be usefully applied to many more types of behavioral data. For example, in cognitive studies, the probability of, and time to learn an association could be modeled. Such approaches could contribute to understand of learning through statistical modeling of the age at which a behavior is first performed or develops, or the number of trials taken for a response to be learned or extinguished. Similarly, MSM could be usefully applied to a wide range of behavioral data on short- and long-term changes in state. Much behavioral research has focused on the choices animals make and particularly how the current choice might depend on previous choice(s). Such choices and the state dependency of choices (28) could be modeled using multistate modeling.

An important consideration when undertaking any statistical analysis is the independence of each data point in a dataset; this is an underlying assumption of many statistical tests. A lack of independence between points can lead to pseudo-replication of results and falsely low *p*-values for explanatory variables. Frequently, in animal behavior data, there may be reasons for similarity between data points; because data are collected from the same animal, from animals from the same location (e.g., pen or farm), or between animals with genetic similarities (e.g., siblings or half siblings). This leads to correlations between data points in a dataset that need to be accounted for (e.g., using random effects terms), to understand the true influence of explanatory variables (4, 29, 30). In this study, we considered the effect of Dam and Assessor as frailty terms, which are in essence random effects. The “Dam” effect accounts for the genetic influence of each dam (i.e., accounts for genetic similarities between litter mates) and also elements of each dam’s specific environment; these aspects were identified as important potential sources of correlation in our dataset. Assessor was included to account for variability in interpretation in behavior between different individuals but was not required in models, presumably due to the questionnaire’s high inter-rater reliability (15). However, dam was indeed an importance source of variance in survival models; the influence of this variable in the model altered the significance of explanatory variables in some models. This further illustrates the need to consider random effects and the structure of shared sources of variance in behavioral data analysis.

The two epidemiological statistical approaches used here have some limitations, which it is worth briefly highlighting. The methods typically require more data to reach an adequate power than simpler statistical methods. As with any multivariate analysis, modeling more than one outcome can make interpretation more difficult. There are many different approaches to survival analysis and MSM and such choice can make it difficult to select the most appropriate method. Furthermore, each approach has assumptions and these assumptions can be hard to meet with real data. As models become progressively more complex, model selection and model fit become increasingly important considerations. Finally, it is worth highlighting that these approaches reveal associations and do not reveal causation. For example, in Guide Dogs’ data, the finding that some breeds and dogs not bred by Guide Dogs were withdrawn faster could have resulted from stereotypes in expectations of these dogs based upon breed or source, rather than characteristics of the individual dogs.

## Conclusion

Survival analysis and MSM permit data analysis of a time variable and discrete outcome or outcome(s) together, which are common data types in animal behavior. These methods can be used to provide additional insight to more traditionally used statistics in this area, providing an overview of temporal patterns and reducing multiple testing. Using data from guide dogs on length of time in states of training and working, multistate modeling particularly was useful in understanding the overall system-level patterns and individual differences.

## Data Accessibility

The datasets used in this study are described in elsewhere (references provided in text).

## Ethics Statement

The study was conducted in accordance with ethical guidelines and approval from Guide Dogs, UK, and the University of Nottingham institutional guidelines.

## Author Contributions

LA conceived of the manuscript, supervised design of data collection tools and data collection, conducted statistical analysis, and wrote the original draft manuscript; NH designed original data collection tools, collected data, and helped draft the manuscript; MG helped secure funding, participated in data analysis, and edited the manuscript; GE secured funding, supervised the study, and edited the manuscript.

## Conflict of Interest Statement

Authors of this publication frequently consult with Guide Dogs. Guide Dogs have approved the paper for publication, but have not altered the results or presentation of the results in any way. The terms of this arrangement have been reviewed and approved by the University of Nottingham in accordance with its policies on research.
